# Intimate Partner Violence, Sexual Assault, and Child Abuse Resource Utilization During COVID-19

**DOI:** 10.5811/westjem.2022.4.55582

**Published:** 2022-07-11

**Authors:** Jennifer Pallansch, Claire Milam, Kendra Ham, Patricia Morgan, John Manning, Jessica Salzman, Kathryn Kopec, Margaret Lewis

**Affiliations:** * Indiana University School of Medicine, Department of Emergency Medicine, Indianapolis, Indiana; † Medical University of South Carolina, Department of Emergency Medicine, Charleston, South Carolina; ‡ Levine Children’s Hospital, Atrium Health Carolinas Medical Center, Charlotte, North Carolina

## Abstract

**Introduction:**

Key measures in preventing spread of the virus that causes coronavirus disease 2019 (COVID-19) are social distancing and stay-at-home mandates. These measures along with other stressors have the potential to increase incidences of intimate partner violence (IPV), sexual assault, and child maltreatment.

**Methods:**

We performed a retrospective review of county police dispatches, emergency department (ED) visits, Sexual Assault Nurse Examiner (SANE) consults, Domestic Violence Healthcare Project (DVHP) team consults, and Child Protection Team consults at a large, tertiary, Level I trauma center. We queried International Classification of Diseases Revision 10 codes most specific to IPV, sexual assault, and child maltreatment from March–October 2020 compared to 2019. Similarly, the number of consults performed by SANE, DVHP, and our Child Protection Team were collected. We compared all ED visits and consultations to total ED visits for the reviewed time period. Finally, the total number of calls and referrals to a child advocacy center and resource call line for victims were recorded during this timeframe.

**Results:**

Police dispatches for IPV-related assaults increased by 266 reports from 2019 to 2020 (P = 0.015). Emergency department visits related to IPV increased from 0.11% of visits in 2019 to 0.15% in 2020 (P = 0.032), and DVHP consults increased from 0.31% in 2019 to 0.48% in 2020 of ED visits in the first three months (P < 0.001). Child maltreatment visits increased from 0.47% of visits in 2019 to 0.81% of visits in 2020 (P = 0.028), and a higher percentage of patients required Child Protection team consults from 1% in 2019 to 1.6% in 2020 (P = 0.004). Sexual assault-related visits and SANE consults both showed a small increase that was not statistically significant. Fewer calls and referrals were made to our child advocacy center and resource call line, decreasing by 99 referrals and 252 calls, respectively.

**Conclusion:**

Despite decreased ED volumes throughout the pandemic, we observed an increase in police dispatches, ED visits, and utilization of hospital consult services related to IPV and child maltreatment following the initiation of stay-at-home orders. However, use of community resources, such as the local child advocacy center, declined.

## INTRODUCTION

As of December 2021, the coronavirus disease 2019 (COVID-19) pandemic has affected more than 270 million people and caused more than five million deaths worldwide.^1^ North Carolina has reported greater than 1.5 million cases and 19,000 deaths.^2^ One of the primary mitigation measures to prevent the spread of COVID-19 has been social distancing enforced by a variety of state, county, and city restrictions.^3^,^4^ Mecklenburg County, the largest county by population in North Carolina, issued its stay-at-home order on March 24, 2020, followed shortly with a statewide mandate by the governor on March 27, 2020.^5^–^7^ Under these mandates, people were advised to leave their home only for essential purposes, gatherings were limited to 10 people, childcare centers were open for essential workers only, and most other retail and social services were closed. This was followed by an initial three-phase system of de-escalation of these social restrictions dependent upon the state of the pandemic that began to once again escalate in November 2020.

Social distancing has been an essential component in limiting the spread of the severe acute respiratory syndrome-coronavirus 2 (SARS-CoV-2) virus.^4^ However, given the unprecedented nature of this pandemic, little evidence exists to define the types of services and resources that should be expounded upon or limited in response to this protective measure. Social distancing has the potential to increase the incidence of unseen IPV, sexual assault, and child maltreatment. With stay-at-home orders in place, victims may have limited ability to escape their abusers while also being less visible to mandatory reporters or other sources of support. The transition to virtual school may decrease opportunities for children suffering from abuse or neglect to be recognized or to seek help. In addition, other social stressors contribute to increased risks of violence. Rising unemployment levels, mass hysteria, and documented surges of racism and xenophobia in combination with the mental effects of isolation have resulted in worsening psychologic and financial stressors.^8^ Poverty and financial pressures have been repeatedly demonstrated to correlate with higher rates of child maltreatment and IPV.^9^,^10^

In our study we looked to further clarify the relationship between social distancing, the COVID-19 pandemic, and resource utilization by victims of IPV, sexual assault, and child maltreatment within our community. Notably, Mecklenburg County had the highest number of violent crimes and total crime index reported in North Carolina in 2019.^11^ Historically, this area has also had a high percentage of child maltreatment cases, with 14,233 complaints filed in 2018, comprising nearly 5% of all children in the county.^12^ Despite the local prevalence of violent crime and abuse, several community resources serving victims were closed or downscaled at the beginning of the pandemic, while others shifted their response to a virtual platform and maintained services. However, essential agencies such as police and emergency medical services (EMS) as well as local emergency departments (ED) continued to function throughout the pandemic regardless of the stay-at-home mandates. Considering the multifaceted nature of victim identification and reporting, our study looked to several resources in our community to examine the incidences of reported abuse and assault during the COVID-19 pandemic.

*Population Health Research Capsule*

What do we already know about this issue?

*Disasters and social isolation can potentially increase the risks of intimate partner violence (IPV), sexual assault, and child abuse in vulnerable populations.*

*What was the research question?*

*We studied resource utilization by victims of IPV, sexual assault, and child abuse in Charlotte, NC, during the COVID-19 pandemic.*

*What was the major finding of this study?*

*We found similar or increased rates of police calls and hospital visits but decreased use of community resources.*

*How does this improve population health?*

*Further consideration is needed during natural disasters and social distancing to account for violence in the home and the ability of victims to access resources.*

## METHODS

Following study approval by the institutional review board, we performed a retrospective review of several prehospital, hospital, and outpatient advocacy centers that provide resources specific to IPV, sexual assault, and child maltreatment. Volumes of visits, calls, and consults in the Charlotte-Mecklenburg County region were compared from 2019 to 2020. We queried records starting from the initial month of declared states of emergencies and stay-at-home mandates through the following seven months and compared data to the same timeframe from the previous year, from March 1–October 31, 2020, and March 1–October 31, 2019. After this time, social distancing mandates again briefly escalated and underwent more rapid and less consistently enforced changes; thus, we looked to capture only the initial response during the pandemic.

We queried dispatch records from the Charlotte-Mecklenburg Police Department (CMPD) for assault during this timeframe. The CMPD assault cases had been specifically filtered per standard CMPD protocols and categorized as related to IPV. The training and criteria for this categorization did not undergo changes during the timeframe of our study.

We reviewed Sexual Assault Nurse Examiner (SANE) consults, Domestic Violence Healthcare Project (DVHP) advocacy team consults, and Child Protection Team consults that took place at a Level 1 trauma center that sees approximately 80,000 adult and 30,000 pediatric patients annually. We saw a significant decrease in the total number of patients presenting to the ED during the pandemic. To account for the overall decreased ED volume during this time, we compared the total number of consults as well as the percentage of patients with an ED visit requiring these services in the delineated timeframe.

Additionally, we examined ED visits at this center related to abuse and sexual assault. Patient encounters were queried with *International Classification of Diseases 10^th^ Revision* (ICD-10) codes specific to IPV, sexual assault, and child maltreatment (Supplemental Table 1). Again, visits were compared relative to total ED volumes and reported as percentages for the time period described above to account for fluctuating patient volumes. The ICD-10 codes and variables were defined as above with case selection criteria discussed and agreed upon among all authors. Authors acted as data abstractors and were trained prior to chart review and therefore were not blinded. Although this study focused on the overall number of patient encounters in the ED coded with ICD-10 codes specific to IPV, sexual assault and child maltreatment, we reviewed a random sample of charts for these encounters to ensure that the charts were properly coded based on clinician documentation. Two abstractors reviewed 15% of charts, and using Cohen’s kappa they then analyzed the charts for inter-rater reliability of the categorization of the ICD-10 code documented in the patient encounter. Except where otherwise reported, data was analyzed using Wilcoxon rank-sum and chi-square analysis with a two-tailed hypothesis, and *P* < 0.05 was considered statistically significant. We held regular meetings to discuss chart review results, and any charts in which it was not clear whether an ICD-10 code under the designated categories was appropriate based on clinician documentation were reviewed by the group and consensus reached.^13^

Finally, the total number of consults and basic demographic information was obtained from two outpatient resources. Investigators were provided deidentified call logs from Safe Alliance’s Greater Charlotte Hopeline. Safe Alliance is a Charlotte-based nonprofit organization that provides resources and counseling for victims of sexual assault and IPV. Additionally, metrics were obtained from Pat’s Place Child Advocacy Center, a child-friendly facility that performs forensic interviews and provides family advocacy services, as well as helps to coordinate investigation, prosecution, and treatment of child maltreatment cases in Mecklenburg County. Pat’s Place accepts referrals from both the Department of Social Services (DSS) as well as law enforcement.

## RESULTS

**Figure 1:**
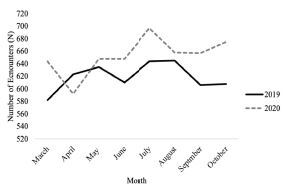
Charlotte-Mecklenburg Police Department encounters for intimate partner violence-related assaults from 2019–2020.

In 2020, there were 5219 reports of IPV documented by the CMPD compared to 4953 reports in 2019 (Figure 1), significantly increasing by 266 reports (*P* = 0.015). As seen in Table 1, the majority of victims were 18–29 years old, Black, and female across both study time periods. The most common charge against the perpetrator was non-aggravated assault followed by aggravated assault, both of which increased in 2020 compared to 2019 (*P* < 0.001 and *P* = 0.005, respectively) (Supplemental Table 2). Increased or similar rates of all types of perpetrator charges were reported with the exception of rape, which modestly decreased in 2020 from 37 reports to 29 (*P* = 0.213). Comparable rates of death, gun threats, and serious injury were reported. Of note, more victims were treated on scene and released (*P* = 0.015). While 77 more people refused treatment in 2020 compared to 2019, these differences were not statistically significant (*P* = 0.798).

**Figure 2:**
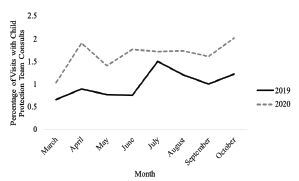
Percentage of emergency department visits with Child Protection Team consults from 2019–2020.

In review of hospital resources, there were significant increases in ED visits leading to hospital admissions requiring Child Protection Team consults from 1% to 1.6% (*P* = 0.004) (Figure 2). The majority of children were White and Black, ages 0–5 (Table 2). There were slightly fewer total numbers of consults, with 182 consults in 2020 compared to 189 consults in 2019. When accounting for the substantial decrease in pediatric ED volumes in 2020, this shows a significant increase in visits requiring Child Protection Team services on average and across every month of the eight-month study period.

**Figure 3:**
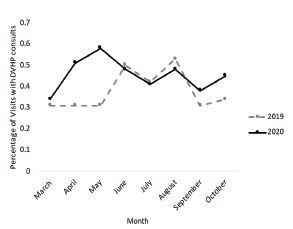
Percentage of emergency department visits with Domestic Violence Healthcare Project team consults from 2019–2020.

**Figure 4:**
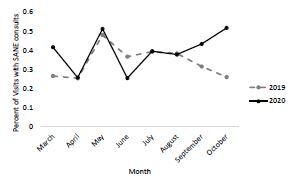
Percentage of emergency department visits with Sexual Assault Nurse Examiner consults from 2019–2020.

Similarly, the full number of consults for DVHP and SANE services decreased over our time frame in 2020, but a higher percentage of patients required DVHP and SANE services when accounting for ED volumes (Figures 3 and 4). However, only the proportional increases in DVHP consults in the first three months were statistically significant, increasing from consulting on an average 0.31% of visits in 2019 to 0.48% of visits in 2020 (*P* < 0.001). Over the full study timeframe, DVHP consults increased from .38% of visits in 2019 to .45% of visits in 2020 (*P* = 0.060). In 2020 SANE was consulted in 0.40% of ED visits compared to 0.34% in 2019, but this was not a significant increase (*P* = 0.226).

**Figure 5:**
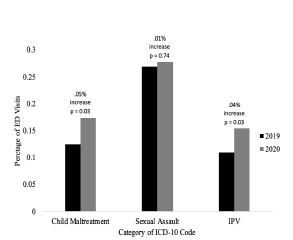
Percentage of emergency department visits with ICD-10* codes related to child maltreatment, sexual assault, and intimate partner violence. *ICD-10, International Classification of Diseases 10th Revision.

When reviewing ICD-10 codes for ED visits, we identified the 41 most applicable codes (Supplemental Table 1). As seen in Figure 5, there were proportional increases in the percentage of ED visits for IPV and child maltreatment during the study timeframe (*P* = 0.031 and *P* = 0.028, respectively). There was also a small, but statistically insignificant, increase in ED visits with ICD-10 codes related to sexual assault (*P* = 0.743). Fifteen percent of the charts were selected, and the ICD-10 codes were reviewed and categorized by a second trained investigator into one of the categories of IPV, sexual assault, child maltreatment, or removal from the study if it was not applicable with perfect agreement, to include 173 of 187 charts. There was disagreement on one chart in the categorization as IPV, sexual assault, or child maltreatment with almost perfect agreement at 99.5% and Cohen’s k = 0.993.

Referrals to Pat’s Place Child Advocacy Center decreased, with 410 referrals in 2019 and 311 referrals in 2020. A higher percentage of cases were referred from DSS compared to law enforcement, at 33% vs 27%, respectively. The Safe Alliance Greater Charlotte Hope Line fielded marginally fewer callers in 2020 compared to 2019, from 6518 to 6770. There was a decrease in primary and secondary call reasons for IPV from 5,059 in 2019 to 4,764 in 2020 (*P* < 0.001). Certain services provided through the hotline increased and included advocacy, caregiver education, court education, crisis intervention, emotional support, legal resource information, prevention, and safety planning compared to the previous year (Supplemental Table 3). Given the multitude of sources with varying trends, the data across each source was consolidated (see Table 3).

## DISCUSSION

The COVID-19 pandemic has had a profound effect on our community and on another ongoing crisis in our nation: IPV and violence in the home. While we know that all persons are at risk of experiencing IPV, those most affected are females of color, as reflected in our CMPD and Safe Alliance data.^14^ We found that Black females aged 18–29 made up the highest proportion of reports of IPV and sexual assault as well as the highest number of calls to the hotline for support.

Social determinants of health affect all facets of life including responses to disasters. The financial and social stresses of rising job instability and losses, childcare, and ability to afford and successfully participate in virtual schooling is poised to have profound effects on victims and abusers. Economic inequalities in a relationship and poverty have been shown to increase risks of IPV.^14^ The pandemic has disproportionately affected women, minorities, immigrants, and workers without a college education, increasing the risks to some of the most vulnerable people in our community.^15^,^16^ Previous literature demonstrated that natural disasters and stay-at-home guidelines increase reports of sexual assault, IPV, and support services needed for victims.^17^,^18^ A review of assault cases in Florida over a nine-year period demonstrated increased assault rates by approximately 78 cases per year during prolonged exposures to natural disasters, defined as >199 days of declared disaster.^18^ Similarly, a review of child maltreatment cases reported after natural disasters Hurricane Hugo, Hurricane Andrew, and the Loma Prieta earthquake found substantial increases for 3–6 months afterward.^19^ However, information from these studies is often limited as there is variability in reporting methods, definitions of abuse, and methodologies as evidenced in a meta-review of child maltreatment reports related to natural disasters in the US, which showed conflicting relationships between natural disasters and child maltreatment.^20^

Our study found consistent increases in utilization of resources for child maltreatment during the COVID-19 pandemic. Both White and Black children were among the highest groups seen by our Child Protection Team, and we saw equitable total numbers of consults and significantly increased percentages of ED visits requiring their services across all months of the pandemic. When looking at ED visits, there were significant increases in the percentage of visits coded as related to child maltreatment. Despite this increase, we saw fewer referrals to Pat’s Place and fewer calls to Safe Alliance for child maltreatment. Referrals to Safe Alliance were made by calling their Hope Line, and it is certainly possible with the stay-at-home mandate that victims had less opportunity to even make a phone call. Changing work hours, closures, and limited staffing in the early portion of the stay-at-home mandate at Pat’s Place and Safe Alliance could have also affected referrals. However, this suggests that across both cases, adults are not engaging with outpatient resources that require calling, and it may be that the lack of ability to get away from abusers affected the ability to access call and support lines. However, this is still a speculative relationship that requires further research.

Within our data, we found similar rates of sexual assault from the previous year, both in police dispatches, SANE consults, and ED visits. Similarly, although we had initial increases in DVHP consults within the first three months of the pandemic, there were not significant changes after this time. The first three months of our study represented the strictest degree of social distancing, with recommendations to leave the house only for essential purposes, thereby providing more contact with abusers, which may have had a greater impact on rates of IPV.

While our hospital consult services saw only early increases in utilization, we saw increasing reports to CMPD of assault related to IPV and ED visits coded as related IPV across the entire study timeframe. This confirms that we are seeing at least similar levels of sexual assault and increased incidence of IPV in our community as we would expect given the unique psychological and financial stressors related to social distancing and the pandemic. Additionally, we know that there has been an overall decrease in patients seeking medical attention during the pandemic, often out of fear of contracting the virus, which may have further decreased utilization of healthcare resources for IPV, sexual assault, and child maltreatment.^21^–^24^ Therefore, it is of particular note that despite well-documented avoidance of healthcare during the early pandemic, we continued to see instances of similar and higher percentages of patient’s presenting to the ED for IPV, child maltreatment, and sexual assault and requiring hospital-specific consult services for victims.

## LIMITATIONS

Our study had several limitations. Specifically, we found variability in documentation and coding of ED visits for possible child maltreatment, IPV, and sexual assault. When evaluating the total number of ED visits requiring DVHP, the Child Protection Team, or SANE consults compared to the number of ED visits with an ICD-10 code specific to these diagnoses, there were fewer ED visits compared to the number of consults, suggesting clinicians are hesitant to include ICD-10 codes indicating abuse. This demonstrates that the ICD-10 codes selected often do not fully describe concerns for assault and abuse and overall limit the ability to include all patients presenting with these complaints. However, as investigators queried the same codes from 2019 to 2020 it was assumed that the same number of patients improperly coded or miscoded would be missed from year to year. Additionally, there had not been hospital-specific training or mandates addressing these discrepancies or changes to documentation; so it is unlikely to have significantly impacted the data. Finally, patients at our ED had access to their patient portal and full chart several years prior to our study, in 2015, and we would not expect patient access to the electronic health record to affect clinician documentation during our study period.

Following chart review, we found that a small number of patient charts coded as IPV actually described elder or familial abuse. It has been previously demonstrated in the literature that caregivers and the elderly have many of the same risks as those experiencing violence from an intimate partner.^25^ As elder and familial abuse is affected by the same stressors as those contributing to other forms of abuse and these patients also require additional, sometimes overlapping resources, we included these charts under the larger umbrella of IPV. However, the extent of elder and familial abuse cases cannot be evaluated through this study, and it is unclear how much it contributed to the significant increases in ED visits for IPV.

Additionally, we had initially planned to include EMS records. However, it was discovered that specific coding for IPV, sexual assault, and child maltreatment does not currently exist and that these cases are categorized into broader, medically focused categories. While the narrative permits prehospital personnel to document occurrences in patient’s words that allowed investigators to reasonably differentiate assault from IPV, this documentation in the narrative was inconsistently performed. Ultimately, assault data from EMS was removed from the study as we could not reliably compare rates of assault specific to IPV. The exception to this is in cases of strangulation where paramedics have the option to select strangulation as a diagnosis within their documentation. While the overall numbers are low, there was an increase in EMS response in which a diagnosis of strangulation was given with three cases reported in 2019 compared to 11 cases in 2020. However, a county-wide initiative involving EMS training specific to strangulation occurred in October 2019; thus, this data was ultimately thought to be too inconclusive to include in the analysis.

We purposefully included “duplicates” in this study, in the sense that we looked to potentially capture the same individual accessing multiple resources from the prehospital, hospital, and community setting. As we are comparing total numbers of calls, consults, and visits only between resources (ie, the total number of police dispatches in 2019 compared to police dispatches in 2020), we did not expect individuals accessing multiple resources to significantly affect our statistical analysis.

A final but critical limitation to recognize is the number of people who have abstained from all medical care and resources and could not be accounted for in our study. Literature has demonstrated that people have delayed and often forgone medical care during the COVID-19 pandemic. One study collected survey responses from 1337 participants and showed that 41% of responders who needed care reported forgoing medical care during this period, primarily out of fear of COVID-19 transmission and financial stresses.^24^ There was a decrease in ED visits by 42% from March 29 to April 25, 2020, across the US, highest among patients who were **≤**14 in age and female; this was particularly relevant to our study, which demonstrated that children and young females were at higher risk for experiencing abuse.^23^ Hospitalizations for acute and life-threatening events such as heart attacks and stroke were markedly decreased in the beginning of the pandemic, which showed that even for life-threatening concerns, patients were avoiding presenting to a hospital.^22^ While a percentage of these cases may be accounted for in the increased police dispatches and more refusal of care and transport to the hospital, the literature suggests there is a portion of the population experiencing abuse and assault injuries that we were unable to account for in this study.

## CONCLUSION

We found increases in intimate partner violence and child maltreatment resource utilization associated with social distancing and the COVID-19 pandemic. Police calls for assault increased by 5.4% from 2019 to 2020. The percentage of ED visits for child maltreatment (0.12% to 0.17%), IPV (0.11% to 0.15%), and sexual assault (0.27% to 0.28%) also increased from 2019 to 2020, respectively, despite an overall decrease in the number of ED visits. These increases in reporting and ED visits were not reflected in the numbers found in our existing community resources. Rather, community resources including Safe Alliance and Pat’s Place Child Advocacy Center saw decreases in the number of calls and referrals.

It is possible that known closures, limitations in staffing, and an inability to contact community resources safely from the home may have affected this increase in hospital and police dispatches associated with a conflicting decrease in community advocacy calls and referrals. However, further work is needed to investigate this relationship to identify and assist those experiencing violence in the home during natural disasters such as the COVID-19 pandemic and to understand how people seek out and identify community resources. The increases in types of violence experienced in the home for those using police and ED resources should be considered in disaster response and hospital planning as our response to the pandemic evolves. With children in virtual school, training for recognition of child maltreatment by teachers via a virtual platform may be needed as well as clear communication regarding availability of community resources and how to access them. Additionally, clinicians on virtual platforms and in the ED should continue to be vigilant for the signs or symptoms of intimate partner violence and child maltreatment.

## Supplementary Information



## Figures and Tables

**Table 1:** Charlotte-Mecklenburg Police Department demographic data for all encounters categorized as intimate partner violence-related assaults from 2019–2020.

Demographics	Cases 2019 (N)	Cases 2020 (N)	P-value
Total	4,953	5,219	*0.015
Age			0.363
18–29	2,122	2,280	
30–39	1,338	1,428	
40–49	823	800	
>50	670	711	
Race			0.413
Amer In/Alaska Nat	3	9	
Asian	41	46	
Black	3,393	3589	
Native Hawaiian	3	5	
White	1,449	1,477	
Unknown	64	95	
Gender			0.556
Male	1,350	1,396	
Female	3,601	3,823	

*Note:*

* significant at P < 0.05.

*Amer In/Alaska Nat*, American Indian/Alaska Native.

**Table 2:** Demographic data for patients with a Child Protection Team consult from 2019–2020.

Demographics	Cases 2019 (N)	Cases 2020 (N)	P-value
Total	189	182	0.833
% of ED Visits	1.0%	1.6%	*0.004
Age			0.820
0–5	149	148	
6–12	15	12	
13–17	25	22	
Race			0.904
Amer Ind	2	4	
Asian	5	5	
Black	68	75	
Latinx	17	18	
White	80	76	
Unknown	5	0	
Gender			0.794
Male	95	89	
Female	93	92	

*Note:*

* significant at P < 0.05.

*ED;* emergency department; Amer Ind, American Indian.

**Table 3:** Summary of trends rates of various types of reports of intimate partner violence, sexual assault, and child maltreatment from 2019 compared to 2020.

Summary table	2019	2020	P-value
IPV			
Police: IPV-related assault (# of dispatches)	4953	5219	*0.015
DVHP consults (% of ED visits)	0.38%	0.45%	0.114
ED: ICD-10 codes (% of ED visits)	0.11%	0.15%	*0.039
Safe Alliance: IPV (# of calls)	5059	4764	*<0.001
Sexual Assault			
Police: IPV-related rape (# of dispatches)	41	33	0.222
SANE consults (% of ED visits)	0.34%	0.40%	0.226
ED: ICD-10 codes (% of ED visits)	0.27%	0.28%	0.785
Safe Alliance: Sexual Assault (# of calls)	649	584	*0.010
Child Maltreatment			
CPT consults (% of ED visits)	1%	1.64%	*<0.001
ED: ICD-10 codes (% of ED visits)	0.12%	0.17%	*0.034
Safe Alliance: Child Maltreatment (# of calls)	148	118	0.056
Pat’s Place (# of referrals)	410	311	*0.156

*Note:*

* significant at P < 0.05.

IPV, intimate partner violence; *DVHP*, Domestic Violence Healthcare Project; *ICD-10*, International Classification of Diseases 10th Revision*; SANE*, Sexual Assault Nurse Examiners; *ED*, emergency department; *CPT*, Child Protection Team.
