# Supplementary material for: Intimate Partner Violence, Sexual Assault, and Child Abuse Resource Utilization During COVID-19
Source: West J Emerg Med. 2022 Jul 11;23(4):589–96. doi: 10.5811/westjem.2022.4.55582 (PMC9391008; doi:10.5811/westjem.2022.4.55582)
Supplement: Supplementary file 1 [file wjem-23-589-s001.docx]

Supplemental Table 1. List of ICD-10 Codes queried in 2019 and 2020 specific to intimate partner violence, sexual assault, and child abuse.

| ICD-10 Code | Diagnosis Description |
| --- | --- |
| IPV |  |
| T76.91XA | Unspecified adult maltreatment, suspected, initial encounter |
| T74.91XA | Unspecified adult maltreatment, confirmed, initial encounter |
| 09A.319 | Physical abuse complicating pregnancy, unspecified |
| O9A.311 | Physical abuse complicating pregnancy, first trimester |
| O9A.312 | Physical abuse complicating pregnancy, second trimester |
| O9A.313 | Physical abuse complicating pregnancy, third trimester |
| 09A.511 | Psychological abuse complicating pregnancy, first trimester |
| 09A.32 | Physical abuse complicating childbirth |
| Z04.71 | Encounter for examination and observation following alleged adult physical abuse |
| T74.31XA | Adult psychological abuse, confirmed, initial encounter |
| T76.11XA | Adult physical abuse, suspected, initial encounter |
| T74.11XA | Adult physical abuse, confirmed, initial encounter |
| T76.01XA | Adult neglect or abandonment, suspected, initial encounter |
| T74.01XA | Adult neglect or abandonment, confirmed, initial encounter |
| Child Abuse |  |
| T76.92XA | Unspecified child maltreatment, suspected, initial encounter |
| T74.92XA | Unspecified child maltreatment, confirmed, initial encounter |
| T74.4XXA | Shaken infant syndrome, initial encounter |
| T74.4XXS | Shaken infant syndrome, sequela |
| T74.92XS | Unspecified child maltreatment, confirmed, sequela |
| Z04.72 | Encounter for examination and observation following alleged child physical abuse |
| T76.32XA | Child psychological abuse, confirmed, initial encounter |
| T76.32XA | Child psychological abuse, suspected, initial encounter |
| T76.12XS | Child physical abuse, suspected, sequela |
| T76.12XA | Child physical abuse, suspected, initial encounter |
| T76.02XA | Child neglect or abandonment, suspected, initial encounter |
| T74.12XA | Child physical abuse, confirmed, initial encounter |
| T74.02XA | Child neglect or abandonment, confirmed, initial encounter |
| Sexual Assault |  |
| O9A.411 | Sexual abuse complicating pregnancy, first trimester |
| O9A.412 | Sexual abuse complicating pregnancy, second trimester |
| O9A.413 | Sexual abuse complicating pregnancy, third trimester |
| O9A.42 | Sexual abuse complicating childbirth |
| Z04.42 | Encounter for examination and observation following alleged child rape |
| Z04.41 | Encounter for examination and observation following alleged adult rape |
| T74.52XA | Child sexual exploitation, confirmed, initial encounter |
| T76.22XD | Child sexual abuse suspected, subsequent encounter |
| T76.22XA | Child sexual abuse, suspected, initial encounter |
| T76.21XA | Adult sexual abuse, suspected, initial encounter |
| T74.21XA | Adult sexual abuse, confirmed, initial encounter |
| T74.21XD | Adult sexual abuse, confirmed, subsequent encounter |
| T74.51XA | Adult forced sexual exploitation, confirmed, initial encounter |
| T76.51XA | Adult forced sexual exploitation, suspected, initial encounter |

Supplemental Table 2. Charlotte-Mecklenburg Police Department reports of type and severity of violence for all encounters categorized as intimate partner violence related assaults from 2019-2020.

| Type and Severity of Violence | Cases 2019 (N) | Cases 2020 (N) | p |
| --- | --- | --- | --- |
| Total Reports | 4953 | 5219 | *0.015 |
| Perpetrator's Charge |  |  |  |
| Aggravated Assault | 713 | 907 | * <0.001 |
| Homicide | 7 | 7 | 0.983 |
| Rape/Attempted Rape | 41 | 33 | 0.213 |
| Non-aggravated Assault | 4133 | 4302 | *0.005 |
| Robbery | 59 | 74 | 0.399 |
| Injury |  |  |  |
| Death | 7 | 7 | 0.918 |
| Serious Injury | 102 | 92 | 0.265 |
| Gun Threat | 100 | 126 | 0.183 |
| Minor Injury | 2424 | 2463 | 0.141 |
| Threatened | 527 | 471 | *0.005 |
| No Threat/Injury | 1658 | 1928 | *<0.001 |
| Not reported | 135 | 132 | 0.519 |
| Medical Treatment |  |  |  |
| Pronounced | 5 | 4 | 0.670 |
| Hospitalized | 323 | 300 | 0.813 |
| Treated on Scene/Treated  and Released | 386 | 481 | *0.015 |
| Refused Treatment | 1043 | 1120 | 0.798 |
| Not Treated | 1620 | 1531 | *<0.001 |

Note: * significant at p < 0.05.

Supplemental Table 3. Demographics, reason for call, and services provided for Safe Alliance Greater Charlotte Hope Line calls in 2019 and 2020.

| Demographics | Cases 2019 (N) | Cases 2020 (N) | p |
| --- | --- | --- | --- |
| Total calls | 6770 | 6518 | 0.562 |
| Age |  |  |  |
| 0-18 | 48 | 99 | *<0.001 |
| 18 and up | 3615 | 3932 | *<0.001 |
| Unknown | 3107 | 2487 | *<0.001 |
| Race |  |  |  |
| Amer Ind/Nat Am | 11 | 5 | 0.154 |
| Asian | 29 | 41 | 0.110 |
| Black | 1105 | 1194 | *0.002 |
| Other | 83 | 149 | *<0.001 |
| White | 506 | 594 | *<0.001 |
| Unknown | 5036 | 4535 | *<0.001 |
| Gender |  |  |  |
| Male | 382 | 409 | 0.124 |
| Female | 3601 | 4080 | *<0.001 |
| Transgender | 12 | 7 | 0.287 |
| Unknown | 2775 | 2022 | *<0.001 |
| Primary Reason for Call |  |  |  |
| Domestic Violence | 4567 | 4339 | 0.328 |
| Adult Sexual Assault | 409 | 319 | *0.004 |
| Marital/Date Rape | 18 | 7 | *0.035 |
| Child Physical Abuse | 26 | 22 | 0.655 |
| Child Neglect | 12 | 7 | 0.287 |
| Child Sex Abuse | 92 | 66 | 0.066 |
| Advocacy | 2705 | 5684 | *<0.001 |
| Community Res | 2008 | 1991 | 0.266 |
| Services Provided |  |  |  |
| Crisis Intervention | 1800 | 2999 | *<0.001 |
| Emotional Support | 4309 | 4538 | *<0.001 |
| Safety Planning | 4341 | 5148 | *<0.001 |
| Prevention | 868 | 1911 | *<0.001 |
| Legal Resources | 587 | 1087 | *<0.001 |
| Court Education | 603 | 753 | *<0.001 |

Note: * significant at p < 0.05.
